# Assessing IRS performance in a gender-integrated vector control programme on Bioko Island, Equatorial Guinea, 2010–2021

**DOI:** 10.1186/s12936-023-04755-4

**Published:** 2023-10-25

**Authors:** Kylie R. DeBoer, Liberato Motobe Vaz, Teresa Ayingono Ondo Mfumu, Jose Antonio Mba Nlang, Lucas Ondo, Matilde Riloha Rivas, Sandra Incardona, John Pollock, Michael E. von Fricken, Jeremías Nzamio Mba Eyono, Olivier T. Donfack, Carlos A. Guerra, Guillermo A. García

**Affiliations:** 1 MCD Global Health, Silver Spring, MD USA; 2 MCD Global Health, Malabo, Equatorial Guinea; 3 Ministry of Health and Social Welfare, National Malaria Control Programme, Malabo, Equatorial Guinea; 4 MCD Global Health, Hallowell, ME USA; 5 Department of Environmental and Global Health, University of Florida, Gainesville, FL USA

**Keywords:** Gender equity, Gender equality, Indoor Residual Spraying (IRS), Malaria, Vector control, Equatorial Guinea (Bioko)

## Abstract

**Background:**

Indoor residual spraying (IRS) is a common vector control strategy in countries with high malaria burden. Historically, social norms have prevented women from working in IRS programmes. The Bioko Island Malaria Elimination Project has actively sought to reduce gender inequality in malaria control operations for many years by promoting women’s participation in IRS.

**Methods:**

This study investigated the progress of female engagement and compared spray productivity by gender from 2010 to 2021, using inferential tests and multivariable regression. Spray productivity was measured by rooms sprayed by spray operator per day (RSOD), houses sprayed by spray operator per day (HSOD), and the daily productivity ratio (DPR), defined as the ratio of RSOD to HSOD, which standardized productivity by house size.

**Results:**

The percentage of women participating in IRS has increased over time. The difference in DPR comparing male and female spray operators was only statistically significant (p < 0.05) for two rounds, where the value was higher for women compared to men. Regression analyses showed marginal, significant differences in DPR between men and women, but beta coefficients were extremely small and thus not indicative of a measurable effect of gender on operational performance.

**Conclusions:**

The quantitative analyses of spray productivity are counter to stigmatizing beliefs that women are less capable than male counterparts during IRS spray rounds. The findings from this research support the participation of women in IRS campaigns, and a renewed effort to implement equitable policies and practices that intentionally engage women in vector control activities.

**Supplementary Information:**

The online version contains supplementary material available at 10.1186/s12936-023-04755-4.

## Background

Malaria remains a leading cause of morbidity and mortality in sub-Saharan Africa [1, 2]. National Malaria Control Programmes (NMCP) and Elimination Programmes have led efforts to reduce malaria's impact on health systems and economies in many countries [3], relying on vector control, active case management, chemoprevention, and surveillance [4]. Insecticide-treated nets, larval source management, and indoor residual spraying (IRS) are all components of integrated vector control [5]. Bioko Island, Equatorial Guinea, has relied heavily on IRS as part of a larger effort to control and eliminate malaria since 2004.

Adequate IRS implementation at the household level is crucial for effective vector control. Beyond being physically demanding and laborious work, IRS requires rigorous and orderly application of insecticides to ensure total coverage and effective community protection. Spray operators (SO) must go from house to house, seeking permission from the residents to enter and spray all indoor surfaces that an anopheline mosquito might encounter [6]. Furniture and personal belongings must be moved and covered with protective cloth so that the SOs can easily reach all surfaces. The insecticides and equipment are heavy: the standard 3-gallon IRS pump weighs 15 kgs when full and 7 kgs when empty. During a typical day of spraying, SOs apply a full tank of insecticide. The combination of long days, high humidity, hot temperatures, and occupational exposure to pesticides, requires additional mitigating measures to reduce potential health risks [7].

IRS programmes can struggle to achieve high coverage due to variation in household acceptance of spraying. After years of spraying, household members may become fatigued or be swayed by visitors or other family members to refuse IRS. Bioko Island is also home to a very mobile population; thus, many residents relocate yearly, complicating perennial coverage. IRS may be less effective if community acceptability is low or if walls are not adequately sprayed [8]. Interactions with SOs and perceptions of intervention quality are the main reasons households refuse spraying [9]. If families continue to see mosquitoes inside after IRS, they may become distrustful of IRS as an effective vector control tool, refusing future spraying campaigns and encouraging others to do the same [10]. Hence, competent SOs who thoroughly apply the correct dosage of insecticide to all appropriate surfaces maximize IRS service delivery.

Due to the physical demands of the work and stigmatizing sociocultural factors, women have been largely absent from positions in IRS programmes in sub-Saharan Africa [11, 12]. The lack of research on programmes involving female SOs or policies promoting gender equality and equity in vector control reveals a significant knowledge gap. Gender equality refers to equal access to opportunities and resources for people identified by any gender, while equity is the process by which equality is attained through recognizing historic disadvantages and the particular needs of individuals [13]. It is also important to acknowledge that while gender is not binary, male and female gender constructs are used for analysis in this article because these are the two identities prominently used in sub-Saharan Africa. Given the persisting stereotype that women are less efficient sprayers than men, this disparity is especially pertinent [14].

The current study aims to address the knowledge gap by examining the experience of the Bioko Island Malaria Elimination Project (BIMEP). The BIMEP has prioritized women’s inclusion in its spraying campaigns [15] and has created guidelines to encourage their participation and advancement. Measures include equitable wages, safe and discreet workspaces for women, pregnancy testing protocols for female SOs, health and safety campaigns, and income-generating options during maternity leave. Personal protective equipment is specifically ordered in different sizes and styles to provide safe and comfortable options for all SOs. Furthermore, the programme promotes women's leadership and offers professional development, encouraging female SOs to apply for supervisory positions. This study: (1) quantifies the level of female employment in IRS, (2) investigates potential differences in spray productivity between male and female SOs and (3) discusses the BIMEP's gender-inclusive vector control practices.

## Methods

### Study population and duration

Indoor residual spraying takes place in houses across Bioko Island, the largest island of Equatorial Guinea with an area of about 2000 km^2^. The population is urban, with 85% of the island’s 270,000 inhabitants living in the capital of Malabo [16]. Malaria is endemic with perennial transmission on Bioko; before project implementation the *Plasmodium falciparum* parasite rate was 45% in 2–14 year-old children and has since declined by about 75% [16].

This study included 466 BIMEP SOs from the sixteen spray rounds between 2010 and 2021. Fifteen were excluded due to missing gender or spray productivity data (those who only worked two weeks or less), resulting in 451 SOs included within the analyses. Spray rounds had a median duration of 103 working days, ranging from one to 6 months. Gender-based spray productivity analyses were completed for each spray round during the study period.

### Data source

The study used data from the BIMEP and the NMCP’s IRS operations on Bioko Island, Equatorial Guinea, from 2010 to 2021. The data were obtained from BIMEP's spatial decision support system [17], which has been used previously to assess other spray productivity factors [18].

### Spray productivity data

Spray productivity was quantified by the number of houses sprayed by SO per day (HSOD) and the number of rooms sprayed by SO per day (RSOD). A house was recorded as sprayed if at least one room was sprayed. Not every room can be sprayed in all houses due to inaccessible spaces (i.e. kitchens with exposed food) and unsuitable wall surfaces (i.e. ceramic or glass tiles). In some cases, not all sprayable rooms are sprayed because an individual refuses the intervention in certain areas of the house. To account for variation in household sizes and room numbers, the daily productivity ratio (DPR) standardized productivity measures by dividing RSOD by HSOD. On Bioko, a typical household consists of four rooms, and the analysis was limited to residences with fewer than 13 rooms due to the wide range of living conditions.

### SO data

The study examined a range of factors that might influence spray productivity, including age, education, attendance, and longevity at BIMEP. SO data were linked to each spray round through unique, non-identifiable codes. Ethnicity was initially considered as a potential variable due to Bioko Island's diverse ethnic groups. However, no statistical significance was found, and the analysis did not include ethnicity. Unfortunately, the project only had records for demographic data, including age and education, from 2015 onwards. Thus, data for these variables were only available for spray rounds from 2015 to 2021.

Age was divided into three groups: under 25, 25–34, 35 + . Education was ordinally categorized into primary school, lower secondary, upper secondary, and higher education levels. Attendance was calculated by dividing a SO's days worked by each spraying round's days. Interquartile range cut-off values were used to classify attendance as optimal, acceptable, or low. A SO's total rounds worked determined longevity. To account for inadvertent miscoding and workers who departed the programme, a round was counted only if the individual worked more than 2 weeks. Longevity was divided into four roughly equal groups: 1–2 rounds, 3–6 rounds, 7–9 rounds, and 10 + rounds.

### Statistical analysis

All descriptive and multivariable analyses were conducted using R (Version 1.3.1073) [19] packages: broom [20], flextable [21], gtsummary [22], janitor [23], knitr [24], openxlsx [25], scales [26], spatstat.utils [27], and tidyverse [28]. All tests with p < 0.05 were deemed significant. A binomial test assessed whether the proportion of female and male SOs differed significantly for each spray round. Two-sample t-tests determined if each productivity measure (RSOD, HSOD, and DPR) differed by gender. Chi-squared tests, or Fisher's exact tests when assumptions were not met, examined gender differences in age, attendance, education, and longevity. Kruskal–Wallis rank sum tests looked for significant differences in spray productivity by age, attendance, education, and longevity. Linear regression explored gender, attendance, and longevity's univariable associations with spray productivity. These factors were included in two multivariable regression models for 2010–2021, without age and education, and 2015–2021, including age and education.

#### Ethics

This study did not require Institutional Review Board approval because data were collected during normal vector control programme operations and were de-identified before secondary analyses.

## Results

### Gender distribution

**Fig. 1:**
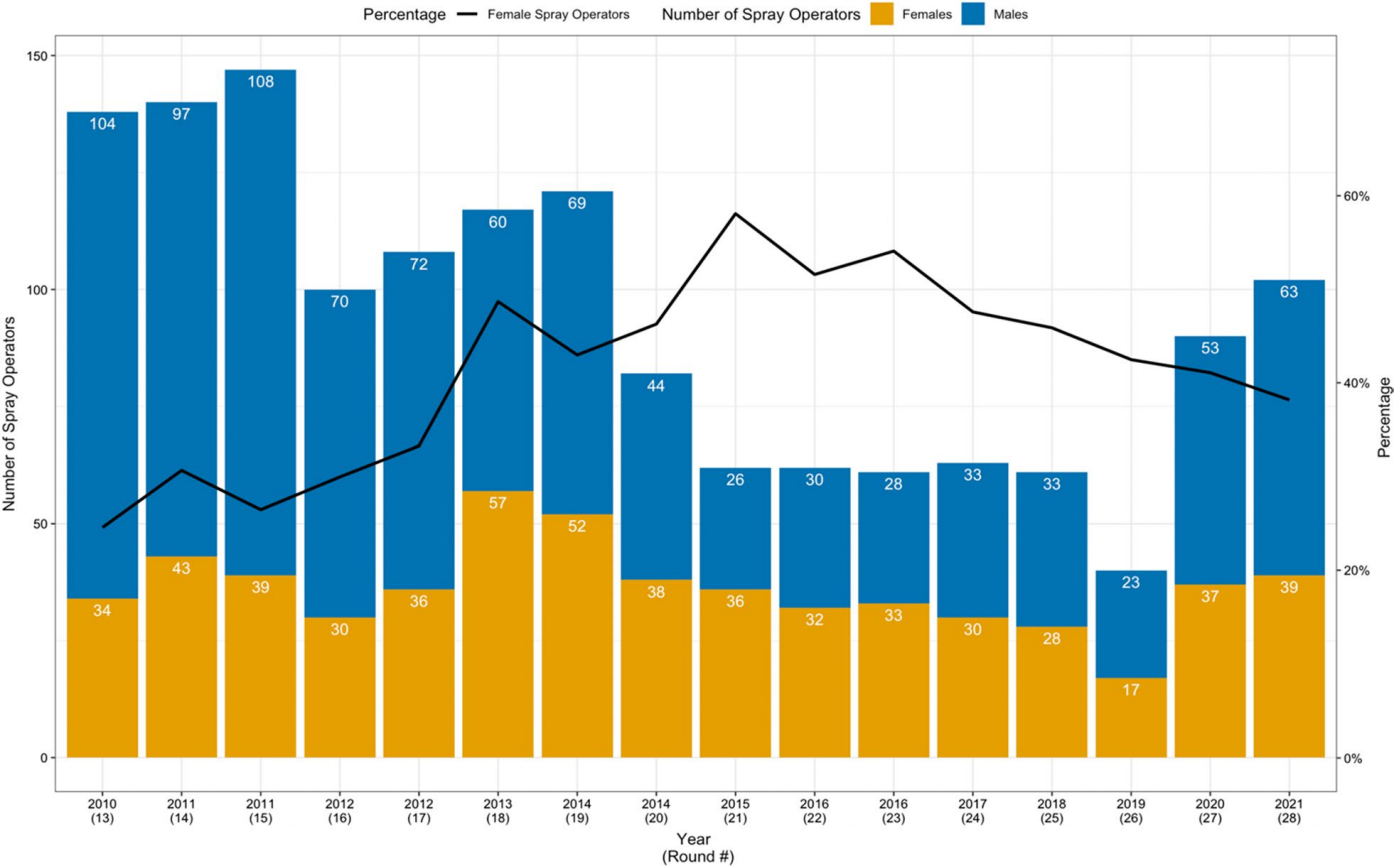
Number and percentage of women spray operators, 2010–2021 (rounds 13–28)

Among the 451 SOs analyzed 147 (32.6%) were women and 304 (66.4%) were men. The workforce ranged from 40 to 147 SOs across rounds with a median of 95 (Fig. 1 and Table 1). Women participated in multiple rounds more frequently than men. The participation of women varied from 24.6% in 2010 (round 13) to 58.1% in 2015 (round 21; Fig. 1). Women comprised most of the workforce in three of the 16 rounds (rounds 21, 22, and 23 in 2015 and 2016). From 2013 to 2020, there was no significant gender difference in the workforce (p $>$ 0.05). More men worked between 2010 and 2012, and in 2021 38.2% of SOs were women. Despite a non-significant decrease in the last round, the proportion of women in the workforce in 2021 was significantly higher than the first round (2010; p < 0.05).

**Table 1:** Descriptive statistics for covariates, overall and by gender for 2010–2021 (rounds 13–28) and 2015–2021 (rounds 21–28)

Characteristics	2010–2021 (Rounds 13–28)		
	Total N	Female N	Male N
Spray operators	451	147	304
Longevity			
Attendance (%)^a^	89.24 (15.18)	89.29 (13.48)	89.19 (16.12)
1–2 Rounds worked	264 (58.54%)	75 (51.02%)	189 (62.17%)
3–6 Rounds worked	130 (28.82%)	43 (29.25%)	87 (28.62%)
7–9 Rounds worked	25 (5.54%)	13 (8.84%)	12 (3.95%)
10 + Rounds worked	32 (7.1%)	16 (10.88%)	16 (5.26%)

	2015–2021 (Rounds 21–28)		
	Total N	Female N	Male N
Spray operators	157	60	97
Age, in years^ab^	32.0 (7.0)	33.0 (9.0)	31.0 (6.5)
Education level^bc^			
Primary school	21 (13.38%)	13 (21.67%)	8 (8.25%)
Lower secondary	101 (64.33%)	39 (65%)	62 (63.92%)
Upper secondary	14 (8.92%)	4 (6.67%)	10 (10.31%)
Higher education	19 (12.1%)	3 (5.0%)	16 (16.49%)

N denotes number

^a^Denotes calculation of median (interquartile range)

^b^Data only available for 2015–2021 (rounds 21–28)

^c^Counts and proportions account for missing data for two individuals lacking education data, one female and one male

### Spray productivity

Table 2 shows the average daily productivity by gender for RSOD, HSOD and DPR in each spray round. Throughout the study, women sprayed 14.52 rooms and 3.77 houses per day, averaging 3.87 DPR, and men sprayed 14.77 rooms and 3.91 houses per day, averaging 3.79 DPR. Gender comparisons by spray round showed no statistically significant differences in average RSOD and HSOD. Except for the two rounds in 2011 (rounds 14 and 15), the average DPR did not differ significantly.

**Table 2:** Average daily productivity measured by RSOD, HSOD, and DPR(RSOD/HSOD) and gender of spray operators, 2010–2021 (rounds 13–28)

Year (R#)	RSOD				HSOD				DPR			
	Avg	Female avg	Male avg	Diff. (95% CI)	Avg	Female avg	Male avg	Diff. (95% CI)	Avg	Female avg	Male avg	Diff. (95% CI)
2010 (13)	17.99	18.27	17.9	0.38 (− 0.94, 1.69)	4.76	4.74	4.77	− 0.03 (− 0.27, 0.21)	3.77	3.87	3.74	0.13 (− 0.09, 0.35)
2011 (14)	15.47	15.94	15.26	0.68 (− 0.44, 1.8)	4.37	4.22	4.44	− 0.22 (− 0.53, 0.1)	3.57	3.82	3.47	0.35 (0.13, 0.57)*
2011 (15)	15.16	15.69	14.97	0.71 (− 0.3, 1.73)	3.85	3.83	3.86	− 0.03 (− 0.25, 0.19)	3.94	4.12	3.87	0.25 (0.07, 0.42)*
2012 (16)	14.6	14.55	14.62	− 0.07 (− 1.32, 1.18)	3.91	3.81	3.95	− 0.15 (− 0.36, 0.07)	3.72	3.81	3.68	0.13 (− 0.08, 0.34)
2012 (17)	14.86	14.81	14.89	− 0.07 (− 1.23, 1.08)	4.08	3.96	4.14	− 0.18 (− 0.39, 0.02)	3.64	3.74	3.59	0.15 (− 0.05, 0.36)
2013 (18)	13.78	13.9	13.65	0.25 (− 0.51, 1.0)	3.87	3.91	3.82	0.09 (− 0.07, 0.26)	3.57	3.56	3.58	− 0.02 (− 0.16, 0.13)
2014 (19)	11.37	11.36	11.37	− 0.02 (− 0.82, 0.78)	2.76	2.75	2.76	− 0.01 (− 0.13, 0.11)	4.11	4.11	4.10	0.01 (− 0.17, 0.2)
2014 (20)	14.11	14.37	13.88	0.49 (− 0.95, 1.92)	3.33	3.33	3.33	0.0 (− 0.16, 0.16)	4.21	4.28	4.15	0.13 (− 0.2, 0.45)
2015 (21)	13.25	13.02	13.58	− 0.56 (− 1.54, 0.42)	3.29	3.25	3.35	− 0.1 (− 0.28, 0.08)	4.02	4.01	4.05	− 0.04 (− 0.23, 0.16)
2016 (22)	15.19	15.03	15.36	− 0.33 (− 1.13, 0.48)	3.99	3.98	4	− 0.02 (− 0.19, 0.15)	3.82	3.79	3.85	− 0.06 (− 0.24, 0.12)
2016 (23)	12.54	12.57	12.5	0.07 (− 0.69, 0.83)	3.38	3.39	3.36	0.03 (− 0.17, 0.23)	3.72	3.72	3.73	− 0.01 (− 0.18, 0.16)
2017 (24)	16.88	17.08	16.7	0.39 (− 0.74, 1.52)	4.44	4.52	4.36	0.15 (− 0.1, 0.4)	3.81	3.79	3.83	− 0.04 (− 0.22, 0.13)
2018 (25)	16.03	16.2	15.88	0.32 (− 0.93, 1.58)	4.05	4.13	3.99	0.14 (− 0.16, 0.45)	3.96	3.93	4.0	− 0.07 (− 0.25,0.11)
2019 (26)	14.75	14.64	14.83	− 0.19 (− 1.38, 1)	3.86	3.8	3.9	− 0.1 (− 0.34, 0.14)	3.82	3.86	3.8	0.05 (− 0.15, 0.26)
2020 (27)	12.25	11.97	12.45	− 0.48 (− 1.36, 0.4)	3.2	3.17	3.22	− 0.05 (− 0.26, 0.15)	3.83	3.79	3.86	− 0.08 (− 0.23, 0.08)
2021 (28)	15.41	15.27	15.5	− 0.23 (− 0.98, 0.51)	3.97	4.03	3.93	0.10 (− 0.06, 0.26)	3.89	3.8	3.95	− 0.15 (− 0.3, 0.00)
Overall	14.67	14.52	14.77	− 0.25 (− 0.58, 0.07)	3.85	3.77	3.91	− 0.14 (− 0.22,− 0.07)*	3.83	3.87	3.79	0.08 (0.03, 0.13)*

^*^ Denotes statistical significance of difference between males and females (p < 0.05)

R# signifies the round number

### Regression analyses

**Fig. 2:**
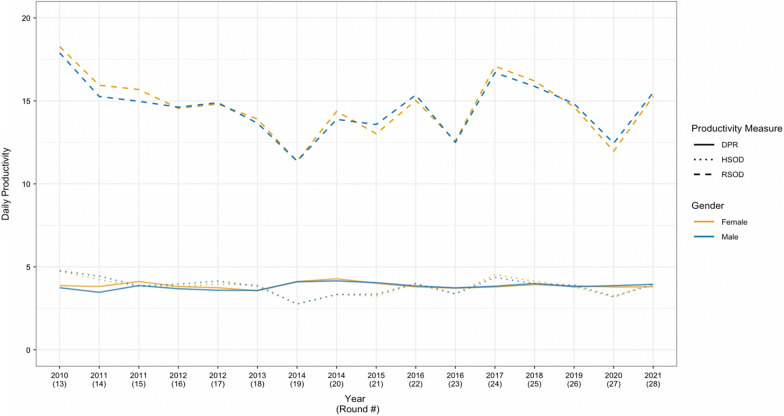
Average daily productivity measured by RSOD, HSOD, and DPR (RSOD/HSOD) by gender of spray operators, 2010–2021 (rounds 13–28). The three productivity measures for females are indicated by orange-yellow graph lines, while the productivity measures for males are indicated by blue graph lines

As depicted in Fig. 2, productivity as measured by RSOD and HSOD varied greatly due to differences in household room sizes. Hence, DPR was used in regression analyses (Additional file 1 and 4).

#### 2010–2021 Spraying rounds model

**Fig. 3:**
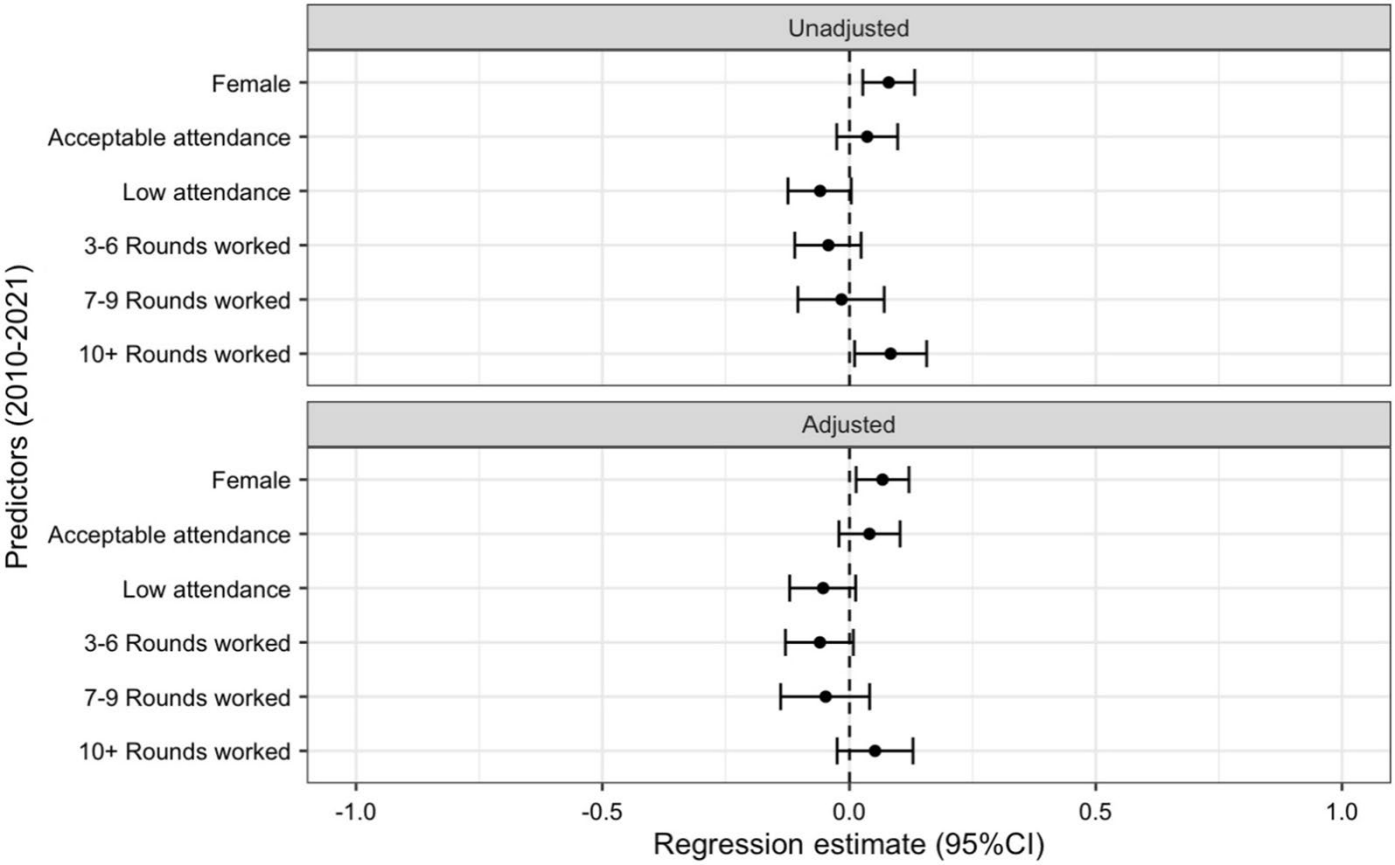
Regression estimates and 95% CIs of productivity (DPR) and gender, attendance, and longevity, 2010–2021 (rounds 13–28). The reference group for each variable is as follows: male, optimal attendance, and 1–2 rounds worked

A gender-related difference in DPR towards higher productivity for women was demonstrated in both the unadjusted model (b = 0.08, p = 0.003) and the multiple linear regression model that accounted for attendance and longevity (b = 0.07, p = 0.014). The exploratory models for RSOD and HSOD can be found in Additional file 2: Fig. S2 and Additional file 3: Fig. S3, respectively. Figure 3 compares productivity with gender, attendance, and longevity in the unadjusted models compared to the adjusted model.

#### 2015–2021 Spraying rounds model

**Fig. 4:**
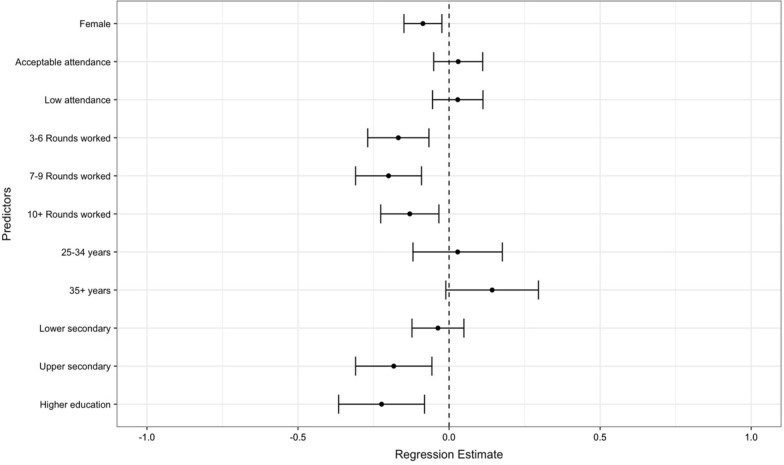
Adjusted estimates and 95% CIs of productivity (DPR) associations with factors, 2015–2021 (rounds 21–28). The reference group for each factor is as follows: male, optimal attendance, 1–2 rounds worked, under 25 years, and primary school

For spray rounds between 2015 and 2021 (rounds 21–28; Fig. 4), age and education were incorporated into the regression model. When adjusting for these covariates, gender had a statistically significant effect on DPR in the opposite direction (refer to Additional file 4: Fig. S4; b = − 0.09, p = 0.007). When compared to SOs with primary school education, SOs with upper secondary or higher education had lower productivity. Compared to the reference group (1–2 rounds worked), longevity demonstrated a statistically significant negative correlation with productivity across all categories. The exploratory models for RSOD and HSOD are in Additional file 5: Fig. S5 and Additional file 6: Fig. S6, respectively.

## Discussion

Gender equity and equality within vector control workforces are critical to the success and sustainability of malaria control and elimination programmes. Evidence suggests that vector control programmes that engage female workers reap direct benefits [2, 29, 31]. Gender-integrated programmes have shown higher coverage rates, and other studies have observed greater household acceptance of interventions when they are conducted by female SOs. Moreover, gender inclusion can promote financial freedom for women, which has been linked to reduced rates of malaria [2, 30]. Gender equity in the workplace recognizes and caters for the unique needs and situations of women, enabling them to contribute meaningfully and feel belonging within the community. Gender equality in the workplace enhances overall success and performance, as women bring diverse experiences and worldviews to the workplace [14]. Some studies have shown that women are more proactive in educating their families and communities about malaria risks and vector control benefits [31]. They are also more adept at exchanging information, creating rapport in social groups, and serving as credible sources of malaria prevention information for other women [32, 33]. As a result of these social connections, female SOs can leverage these networks to boost the impact and reach of IRS [34]. The BIMEP's consistent initiatives to promote women's participation in IRS have aided in improving gender balance in the workforce, offering opportunities for both employment and education.

The impact of workplace gender equity extends beyond malaria and vector control. Encouraging female employment and education aligns with the Sustainable Development Goal 5: Achieve gender equality and empower all women and girls [14]. Employing women boosts economic opportunity and decision-making power [33]. This creates a supportive community of women who inspire future generations. Investments focused on gender not only offer inherent benefits, but also bolster healthcare access and accelerate the reduction of disease burdens [35]. As primary caregivers in most cultures, women are more likely to spend earnings on health and nutrition, improving outcomes from HIV prevention to childhood mortality reduction to health facility deliveries [30, 36, 37]. Despite these benefits, the prevalence of male-dominated programmes and occupations perpetuates the myth that women are less capable.

This study analyzes spray productivity by gender to fill the evidence gap on women's participation in vector control [38]. The study suggests that female SOs on Bioko Island performed IRS duties as successfully as men. In fact, women had marginally higher output than men in the two highest staffing rounds where the DPR difference achieved statistical significance. Women remained on the team during staff reduction rounds (2015–2019) without decline in productivity. Regression models indicated statistically significant but inconsequential DPR differences. The effect size is small; from the 2015–2021 model, it translates operationally to a spray productivity difference of approximately 20 less houses per round for women. This study supports the concept that increasing female participation does not compromise the overall productivity of vector control, but rather guarantees and promotes workforce equity.

In the regression models, several other factors emerged as operationally relevant. While productivity was not statistically significant in any age group compared to those under 25, older SOs presumably possess higher maturity and experience that could improve their communication skills and community rapport. Contrary to initial expectations, sub-optimal attendance did not correlate with reduced productivity. Perhaps surprisingly, SOs with upper secondary or higher education had lower productivity than SOs with primary school education. A plausible explanation could be that advanced degree holders may feel overqualified for IRS, a job often perceived as entry-level requiring extensive manual effort. This insight has operational significance because it emphasizes the importance of leveraging the diverse skill sets and training of employees, especially when planning for professional advancement opportunities. While productivity was lower for those who worked 3–6, 7–9, or 10 or more rounds compared to those who worked 1–2 rounds, this does not account for the operational benefit of workers with prior training and experience. Despite potential burnout, spray operators, mostly women, returned to work additional rounds. Given the physical demand of jobs like IRS, prioritizing rest days and cross-training is essential to consider for long-term sustainability. These operational findings apply to Bioko Island spraying and vector control management.

Few vector control programmes are reported to have gender equitable policies like the BIMEP [14], which includes gender-equal compensation, safe and private changing rooms for women, monitoring SOs for pregnancy due to health and safety concerns and offering alternative jobs for women during maternity leave. Due to privacy and data accessibility constraints, this study did not specifically account for maternity leave; however, anecdotal information indicated it was infrequent. If this influenced the analyses, female productivity would have been underestimated.

The U.S. President’s Malaria Initiative (PMI) actively promoted female engagement and implemented policy reform in malaria control in sub-Saharan Africa since 2015 through the Africa IRS project (AIRS) [14]. The initial work of the PMI AIRS project was promising, and continued female engagement and progress towards gender equity has been taken on by its successor PMI VectorLink. The current paper examined data from a decade of implementation, revealing persistent progress in female engagement, from 24.6% in 2010 to 38.2% in 2021. The study conclusion that the observed differences were not meaningful in the context of programming outcomes is consistent with PMI’s findings. The data suggested household productivity differences comparable to the AIRS project (0.1 to 1.2 households per day) [14] with the absolute difference ranging from as low as 0 and up to 0.22 households per day, depending on the spray round. The analyses used the normalized DPR to account for the heterogeneity in urban and rural household sizes. Rural and urban areas were distributed amongst the workloads for both male and female SOs, thus geographic location was not a contributing factor to differences in number of houses sprayed. Encouraging programmes to take this type of analysis into consideration would provide an additional metric to use allowing for easier comparison of productivity across programmes and countries.

Since 2004, gender-forward policies and female employees have been part of the BIMEP vector control programme. However, these initiatives have not sustained a gender-equitable workforce with female engagement declining from 58% in 2015 to 38% in 2021. The drop in recent years is a call for reinvigorating gender dynamics and engagement in the workforce. The BIMEP actively seeks adaptive management across its activities and these analyses provide an evidence base for reinforcing approaches to gender equality. Policies and mentorship activities can encourage women who are already employees to pursue leadership positions and advance their careers [14, 39], supported by the project’s number of female spray operators participating in other project activities and rising into supervisory roles. However, these measures do not address all the barriers women encounter when entering the workforce, such as unawareness of career opportunities, restrictive cultural norms, domestic obligations, and lack of access to education and role models [39]. As a result of this quantitative research, it is proposed that a new line of inquiry could be developed to elucidate the specific issues and experiences of female SOs on Bioko Island, working in a historically patriarchal society. Qualitative research on social norms could expand the understanding of the programme on how to effectively enhance gender equality within the vector control workforce. Additional insights could be gained by investigating gender differences in SO attitudes, procedure adherence, application rate/quality of application [18], and other factors that influence household acceptability and spray performance.

## Conclusions

All vector control programmes should aim to incorporate gender-inclusive policies and equitable processes, as a step towards achieving comprehensive gender equality. This case study from Bioko Island contradicts prejudiced views that might prevent vector control programmes from actively involving women in the workforce. Targeted recruitment strategies and community education to overcome gender conventions may be effective in engaging more women [39]. Changing cultural attitudes and actively combating implicit biases requires training both staff and community members [33]. The key to improving and maintaining gender equality in vector control programmes hinges on equitable solutions that directly address local hurdles and cultural norms women encounter. If gender-conscious approaches are prioritized, intervention effectiveness can be improved, and health disparities can be reduced. Women represent a highly underutilized demographic within many IRS programmes and must be given equal opportunities to contribute to malaria prevention, control, and elimination efforts.

### Supplementary Information

**Additional file 1: Figure S1.** Model of productivity (DPR) adjusted by gender, attendance, and longevity, 2010–2021.

**Additional file 2: Figure S2.** Model of productivity (RSOD) adjusted by gender, attendance, and longevity, 2010–2021.

**Additional file 3: Figure S3.** Model of productivity (HSOD) adjusted by gender, attendance, and longevity, 2010–2021.

**Additional file 4: Figure S4.** Model of productivity (DPR) adjusted by associated factors, 2015–2021.

**Additional file 5: Figure S5.** Model of productivity (RSOD) adjusted by associated factors, 2015–2021.

**Additional file 6: Figure S6. **Model of productivity (HSOD) adjusted by associated factors, 2015–2021.

## Data Availability

The datasets used and/or analyzed during the current study are available from the corresponding author on reasonable request.

## Abbreviations

AIRS: Africa Indoor Residual Spraying
BIMEP: Bioko Island Malaria Elimination Project
CI: Confidence Interval
DPR: Daily Productivity Ratio
HSOD: Houses sprayed by Spray Operator per Day
IRS: Indoor Residual Spraying
NMCP: National Malaria Control Programme
PMI: President’s Malaria Initiative
RSOD: Rooms sprayed by Spray Operator per Day
SO: Spray Operator
