# Supplementary material for: Assessing IRS performance in a gender-integrated vector control programme on Bioko Island, Equatorial Guinea, 2010–2021
Source: Malar J. 2023 Oct 25;22:323. doi: 10.1186/s12936-023-04755-4 (PMC10599007; doi:10.1186/s12936-023-04755-4)
Supplement: Supplementary file 1 — Additional file 1: Figure S1. Model of productivity (DPR) adjusted by gender, attendance, and longevity, 2010–2021. [file 12936_2023_4755_MOESM1_ESM.pdf]

Characteristic	Beta	95% CI[1]	p-value
Gender			
Male	—	—	
Female	0.07	0.01, 0.12	**0.014**
Attendance			
Optimal	—	—	
Acceptable	0.04	-0.02, 0.10	0.2
Low	-0.05	-0.12, 0.01	0.11
Longevity			
1-2 Rounds worked	—	—	
3-6 Rounds worked	-0.06	-0.13, 0.01	0.082
7-9 Rounds worked	-0.05	-0.14, 0.04	0.3
10+ Rounds worked	0.05	-0.03, 0.13	0.2

[1] CI = Confidence Interval
