# Supplementary material for: Assessing IRS performance in a gender-integrated vector control programme on Bioko Island, Equatorial Guinea, 2010–2021
Source: Malar J. 2023 Oct 25;22:323. doi: 10.1186/s12936-023-04755-4 (PMC10599007; doi:10.1186/s12936-023-04755-4)
Supplement: Supplementary file 2 — Additional file 2: Figure S2. Model of productivity (RSOD) adjusted by gender, attendance, and longevity, 2010–2021. [file 12936_2023_4755_MOESM2_ESM.pdf]

Characteristic	Beta	95% CI[1]	p-value
Gender			
Male	—	—	
Female	-0.22	-0.56, 0.12	0.2
Attendance			
Optimal	—	—	
Acceptable	-0.07	-0.46, 0.32	0.7
Low	0.05	-0.37, 0.47	0.8
Longevity			
1-2 Rounds worked	—	—	
3-6 Rounds worked	-0.41	-0.84, 0.02	0.059
7-9 Rounds worked	-0.89	-1.5, -0.33	**0.002**
10+ Rounds worked	0.04	-0.44, 0.53	0.9

[1] CI = Confidence Interval
