# Supplementary material for: Assessing IRS performance in a gender-integrated vector control programme on Bioko Island, Equatorial Guinea, 2010–2021
Source: Malar J. 2023 Oct 25;22:323. doi: 10.1186/s12936-023-04755-4 (PMC10599007; doi:10.1186/s12936-023-04755-4)
Supplement: Supplementary file 3 — Additional file 3: Figure S3. Model of productivity (HSOD) adjusted by gender, attendance, and longevity, 2010–2021. [file 12936_2023_4755_MOESM3_ESM.pdf]

**Characteristic**	**Beta**	**95% CI**[1]	**p-value**
Gender			
Male	—	—	
Female	-0.12	-0.20, -0.04	**0.003**
Attendance			
Optimal	—	—	
Acceptable	-0.04	-0.14, 0.05	0.4
Low	0.08	-0.02, 0.18	0.11
Longevity			
1-2 Rounds worked	—	—	
3-6 Rounds worked	-0.05	-0.15, 0.06	0.4
7-9 Rounds worked	-0.19	-0.32, -0.05	**0.006**
10+ Rounds worked	-0.05	-0.17, 0.07	0.4

[1] CI = Confidence Interval
