# Supplementary material for: Assessing IRS performance in a gender-integrated vector control programme on Bioko Island, Equatorial Guinea, 2010–2021
Source: Malar J. 2023 Oct 25;22:323. doi: 10.1186/s12936-023-04755-4 (PMC10599007; doi:10.1186/s12936-023-04755-4)
Supplement: Supplementary file 4 — Additional file 4: Figure S4. Model of productivity (DPR) adjusted by associated factors, 2015–2021. [file 12936_2023_4755_MOESM4_ESM.pdf]

Characteristic	Beta	95% CI[1]	p-value
Gender			
Male	—	—	
Female	-0.09	-0.15, -0.02	**0.007**
Attendance			
Optimal	—	—	
Acceptable	0.03	-0.05, 0.11	0.5
Low	0.03	-0.05, 0.11	0.5
Longevity			
1-2 Rounds worked	—	—	
3-6 Rounds worked	-0.17	-0.27, -0.07	**0.001**
7-9 Rounds worked	-0.20	-0.31, -0.09	**<0.001**
10+ Rounds worked	-0.13	-0.23, -0.03	**0.008**
Age Group			
< 25 years	—	—	
25-34 years	0.03	-0.12, 0.18	0.7
35+ years	0.14	-0.01, 0.30	0.068
Education Level			
Primary school	—	—	
Lower secondary	-0.04	-0.12, 0.05	0.4
Upper secondary	-0.18	-0.31, -0.06	**0.005**
Higher education	-0.22	-0.37, -0.08	**0.002**

[1] CI = Confidence Interval
