# Supplementary material for: Assessing IRS performance in a gender-integrated vector control programme on Bioko Island, Equatorial Guinea, 2010–2021
Source: Malar J. 2023 Oct 25;22:323. doi: 10.1186/s12936-023-04755-4 (PMC10599007; doi:10.1186/s12936-023-04755-4)
Supplement: Supplementary file 5 — Additional file 5: Figure S5. Model of productivity (RSOD) adjusted by associated factors, 2015–2021. [file 12936_2023_4755_MOESM5_ESM.pdf]

Characteristic	Beta	95% CI[1]	p-value
Gender			
Male	—	—	
Female	-0.43	-0.85, 0.00	**0.048**
Attendance			
Optimal	—	—	
Acceptable	1.9	1.4, 2.5	**<0.001**
Low	1.0	0.43, 1.6	**<0.001**
Longevity			
1-2 Rounds worked	—	—	
3-6 Rounds worked	-0.10	-0.78, 0.59	0.8
7-9 Rounds worked	-0.10	-0.84, 0.63	0.8
10+ Rounds worked	0.88	0.23, 1.5	**0.008**
Age Group			
< 25 years	—	—	
25-34 years	0.32	-0.68, 1.3	0.5
35+ years	1.0	-0.06, 2.0	0.064
Education Level			
Primary school	—	—	
Lower secondary	0.54	-0.04, 1.1	0.066
Upper secondary	0.34	-0.52, 1.2	0.4
Higher education	0.17	-0.79, 1.1	0.7

[1] CI = Confidence Interval
