# Supplementary material for: Assessing IRS performance in a gender-integrated vector control programme on Bioko Island, Equatorial Guinea, 2010–2021
Source: Malar J. 2023 Oct 25;22:323. doi: 10.1186/s12936-023-04755-4 (PMC10599007; doi:10.1186/s12936-023-04755-4)
Supplement: Supplementary file 6 — Additional file 6: Figure S6. Model of productivity (HSOD) adjusted by associated factors, 2015–2021. [file 12936_2023_4755_MOESM6_ESM.pdf]

Characteristic	Beta	95% CI[1]	p-value
Gender			
Male	—	—	
Female	-0.02	-0.12, 0.08	0.7
Attendance			
Optimal	—	—	
Acceptable	0.47	0.34, 0.60	**<0.001**
Low	0.23	0.10, 0.36	**<0.001**
Longevity			
1-2 Rounds worked	—	—	
3-6 Rounds worked	0.14	-0.02, 0.30	0.094
7-9 Rounds worked	0.16	-0.01, 0.34	0.070
10+ Rounds worked	0.35	0.20, 0.51	**<0.001**
Age Group			
< 25 years	—	—	
25-34 years	0.07	-0.17, 0.31	0.6
35+ years	0.12	-0.12, 0.37	0.3
Education Level			
Primary school	—	—	
Lower secondary	0.17	0.04, 0.31	**0.014**
Upper secondary	0.26	0.06, 0.46	**0.012**
Higher education	0.26	0.04, 0.49	**0.024**

[1] CI = Confidence Interval
